# Protocol-Aware Feature-Based Intrusion Detection System for UAVCAN Networks

**DOI:** 10.3390/s26196061

**Published:** 2026-09-24

**Authors:** Seoyeon Kim, Hyungchul Im, Jongsoo Choi, Seongsoo Lee

**Affiliations:** 1 Department of Intelligent Semiconductors, Soongsil University, Seoul 06978, Republic of Korea; 2 School of AI Convergence, Soongsil University, Seoul 06978, Republic of Korea

**Keywords:** UAVCAN, controller area network, intrusion detection system, protocol-aware intrusion detection, feature extraction

## Abstract

Uncomplicated application-level vehicular computing and networking (UAVCAN) is a higher-layer protocol built on the controller area network (CAN) bus to support control and status communication among electronic control units in unmanned aerial vehicles. Because CAN provides no native authentication or encryption, UAVCAN networks are vulnerable to message injection, replay, and flooding attacks. Most existing CAN intrusion detection systems, however, are designed for automotive networks and do not explicitly account for the transfer structure and tail-byte rules of UAVCAN. This paper proposes a protocol-aware intrusion detection framework that reconstructs UAVCAN transfers and extracts six payload features and four transfer features based on the CAN ID, transfer ID, toggle bit, and transfer-boundary information. The proposed feature set is evaluated using random forest, XGBoost, logistic regression, and multilayer perceptron classifiers on ten attack scenarios from a public UAVCAN dataset. The best-performing configuration achieves average accuracy, precision, and recall of 99.89%, 99.96%, and 99.88%, respectively. Ablation results show that transfer-rule features are particularly effective for replay-attack detection, while combining payload and transfer features provides the most consistent performance across attack types. The proposed approach also outperforms the evaluated automotive CAN IDS baselines and achieves competitive performance compared with existing UAVCAN-specific IDS methods. These findings support the value of protocol-aware feature extraction for UAVCAN intrusion detection.

## 1. Introduction

Unmanned aerial vehicles (UAVs) are being deployed in an increasingly wide range of applications as technology advances and commercialization accelerates, extending beyond traditional military use into civilian and commercial domains [1]. Modern UAVs have evolved from basic flight platforms into intelligent systems that integrate diverse sensing and communication functions. To support real-time control and monitoring, these systems typically comprise multiple electronic control units (ECUs) that operate within an interconnected architecture. In UAV platforms, communication protocols such as micro air vehicle link (MAVLink) and controller area network (CAN) are widely used for data exchange [2,3,4,5,6,7]. In particular, UAVCAN, a higher-layer protocol built on CAN, is used for communication among ECUs in UAV onboard networks.

CAN, which forms the basis of UAVCAN, enables efficient message transmission among ECUs through a broadcast-based serial communication mechanism [8,9,10]. However, CAN does not provide sender authentication or data integrity protection, making it vulnerable to unauthorized access and message injection attacks [11,12,13,14,15]. In addition, because all nodes share the same bus, an attacker who gains access to the network can transmit malicious messages over the bus, thereby disrupting normal network operation. Consequently, attacks such as excessive message transmission, abnormal data insertion, and replay of legitimate messages may occur. These attacks can disrupt normal system control, cause failures in critical system functions, and degrade operational safety.

Owing to these security vulnerabilities, intrusion detection systems (IDSs) for detecting attacks in CAN environments have become an important research topic. In particular, because CAN has long been used as a core communication protocol in in-vehicle networks, numerous studies in the automotive domain have focused on CAN security and IDS design. Among these efforts, machine-learning-based IDSs that exploit the structural characteristics of the CAN protocol and message transmission patterns have advanced considerably. For example, Song et al. proposed a deep convolutional neural network (DCNN)-based IDS that represents CAN IDs at the bit level as two-dimensional frames, achieving high detection performance with a Reduced Inception-ResNet architecture [16]. Hossain et al. developed a long short-term memory (LSTM)-based method using fundamental CAN message fields, including CAN ID, DLC, and payload [17]. To reduce dependence on labeled attack data, a generative adversarial network (GAN)-based intrusion detection system, GIDS, has also been introduced [18]. GIDS extracts only CAN IDs, transforms them into an image-like representation, and detects message injection attacks by learning the distribution of CAN ID sequences in normal traffic.

However, most existing studies have primarily focused on automotive CAN environments and therefore do not sufficiently reflect the unique frame structure and transfer characteristics of UAVCAN. Consequently, such methods may be less effective at detecting security threats that may arise in UAV operating environments, such as flight control disruption, mission failure, and degradation of communication reliability. In UAVCAN, message frames are constructed based on data structures defined in the data structure description language (DSDL). Depending on the transfer payload length, a UAVCAN transfer is carried either in a single CAN frame or across multiple CAN frames. A single-frame transfer carries the entire UAVCAN message within one CAN frame, whereas a multi-frame transfer segments the message across multiple CAN frames. In addition, UAVCAN includes a tail byte at the end of each CAN frame payload to provide frame identification information. This information is used to determine whether frames with the same CAN ID belong to the same transfer and to interpret the frame order within the transfer. Because UAVCAN differs from automotive CAN in both frame composition and transfer behavior, it is difficult to directly apply conventional automotive CAN IDSs to UAVCAN environments.

Accordingly, this study proposes an IDS designed for UAVCAN environments. The proposed method focuses on extracting meaningful payload-level and transfer-rule features by leveraging the structural characteristics of the UAVCAN protocol. Specifically, it uses CAN ID information and transfer-control information contained in the tail byte to extract features that encode UAVCAN protocol rules, and applies these features to several machine learning models to detect attacks. Through this approach, the study evaluates the effectiveness of a feature-extraction strategy that reflects the characteristics of the UAVCAN protocol rather than relying on a specific machine learning model architecture.

The main contributions of this work are summarized as follows:

1. We developed a protocol-aware feature-extraction framework that reconstructs single- and multi-frame UAVCAN transfers and derives six payload-level features and four transfer-rule features from CAN IDs and tail bytes.
2. We conducted an ablation study using four machine learning classifiers with different decision mechanisms to quantify the individual and combined contributions of payload and transfer features.
3. We compared the proposed approach with existing automotive CAN IDS baselines and UAVCAN-specific IDS methods, demonstrating the effectiveness of explicitly modeling UAVCAN transfer rules, particularly for replay attacks.

The remainder of this paper is organized as follows. Section 2 reviews the related work, and Section 3 provides the necessary research background. Section 4 presents a detailed description of the proposed preprocessing and feature extraction methods. Section 5 discusses the experimental results through a comprehensive analysis and addresses the limitations of the research. Finally, Section 6 concludes the paper and outlines directions for future work.

## 2. Related Work

In resource-constrained network environments, communication performance has been actively studied to support stable data transmission. For example, Faheem and Gungor proposed a capacity- and spectrum-aware communication framework for WSN-based smart grid applications [19]. However, the framework mainly focuses on improving communication performance and does not explicitly address intrusion detection or malicious traffic. Due to the inherent security vulnerabilities of the CAN protocol, extensive research has been conducted on intrusion detection in CAN bus environments [20,21,22]. In particular, as attacks on vehicles have increased with the development of autonomous driving technologies, many studies have investigated IDSs for in-vehicle networks by exploiting the structural information and transmission patterns of CAN messages. As a result, machine learning- and deep learning-based IDSs have been developed to detect malicious messages by exploiting not only the fundamental fields of CAN frames but also temporal characteristics such as message periodicity. For example, Desta et al. [23] proposed Rec-CNN, which uses arbitration ID sequences extracted from CAN frames. The method converts consecutive ID sequences into recurrence plot-based images and classifies normal and malicious traffic with a convolutional neural network (CNN). Lo et al. [24] developed HyDL-IDS, a hybrid deep learning-based IDS that incorporates CAN IDs, DLC, and payload information. HyDL-IDS transforms and normalizes the ID, data length code (DLC), and data fields into numerical representations through a CAN traffic preprocessor, and then integrates CNN and LSTM models to learn the spatial and temporal characteristics of network traffic. Ileri et al. [25] introduced MetaCAN, which generates additional features from basic frame information such as CAN ID, DLC, and data fields, including timestamp-based time intervals and ID repetition counts. MetaCAN therefore considers both individual frame fields and inter-message characteristics, including time intervals and ID repetition patterns, to detect various CAN attack types.

Furthermore, several studies have investigated intrusion detection by considering the characteristics of internal CAN communication in UAV environments. Tlili et al. [26] proposed a dynamic intrusion detection framework (DIDF) for UAVCAN environments. DIDF applies an LSTM model to CAN-frame sequences collected from a UAVCAN dataset to learn temporal patterns in network traffic and distinguish between normal and malicious traffic. Du et al. [27] developed UAV-GCNLSTM (UGL), which combines a graph convolutional network (GCN) and an LSTM to account for the limited number of ECUs and the relatively low diversity of information in UAVCAN networks. UGL extracts CAN-frame variation patterns and then constructs a graph representation of the relationships among UAVCAN frames for attack detection. More recently, Mamba-based models have been explored for intrusion detection in Internet of Things (IoT) environments, including UAVCAN-based systems. Wang et al. [28] introduced WaveMamba, which combines wavelet transforms with the Mamba architecture to learn temporal dynamics in UAVCAN traffic from CAN-frame sequences. Although a variety of automotive CAN IDSs and UAVCAN IDSs have been proposed, most existing studies are designed mainly on the basis of CAN frame-level characteristics or statistical patterns and therefore do not directly reflect UAVCAN protocol rules. These differences motivate the development of a protocol-aware IDS that explicitly considers the transfer structure and protocol semantics of UAVCAN.

## 3. Background

### 3.1. Controller Area Network

The CAN protocol supports two frame formats according to the identifier length, namely CAN 2.0A and CAN 2.0B [29]. CAN 2.0A uses the standard format with an 11-bit identifier, while CAN 2.0B uses the extended format with a 29-bit identifier consisting of an 11-bit base identifier and an 18-bit identifier extension. The identifier field of a CAN frame is used for message identification and arbitration. The data field contains the transmitted payload, and its length is specified by the 4-bit DLC in the control field, allowing up to 8 bytes in Classical CAN.

CAN is a widely used serial communication protocol for data exchange among ECUs and employs a multi-master architecture in which multiple ECUs can access the bus without a central controller. In this architecture, any ECU may initiate transmission when the bus is idle. If multiple ECUs attempt to transmit simultaneously, bus access is resolved through CAN ID-based arbitration, and the message with the highest priority is transmitted first. In CAN, logical ‘0’ is defined as a dominant bit, whereas logical ‘1’ is defined as a recessive bit. During arbitration, each ECU transmits its CAN ID one bit at a time while comparing each transmitted bit with the corresponding bus state. If an ECU transmits a recessive bit but detects a dominant bit on the bus, it loses arbitration and stops transmitting. Consequently, the message with the lowest CAN ID wins arbitration and continues transmission. This bitwise arbitration mechanism resolves bus-access contention without corrupting the transmitted frame.

### 3.2. UAVCAN

UAVCAN is a lightweight higher-layer protocol based on CAN 2.0B and is used to exchange control commands and status information among nodes in an internal UAV network. As shown in Figure 1, UAVCAN supports single-frame and multi-frame transfers depending on message size. A single-frame transfer carries an entire message within one CAN frame, whereas in a multi-frame transfer, a message exceeding the payload capacity of a single CAN frame is segmented across multiple CAN frames. In UAVCAN, a transfer is a protocol-level transmission unit used to construct and interpret a message from one or more CAN frames. Accordingly, a single-frame transfer consists of one CAN frame, whereas a multi-frame transfer consists of a sequence of consecutive CAN frames extending from the start-of-transfer (SOT) frame to the end-of-transfer (EOT) frame.

The structure of the UAVCAN tail byte is illustrated in Figure 2. The tail byte is located in the last byte of the data field of each CAN frame and indicates the position and sequential order of the frame within a transfer. It consists of SOT, EOT, toggle bit, and transfer ID, whose functions are described as follows.

- SOT: This bit indicates whether the frame is the beginning of a transfer. It is set to 1 for a single-frame transfer or the first frame of a multi-frame transfer. Otherwise, it is set to 0.
- EOT: This bit indicates whether the frame is the end of a transfer. It is set to 1 for a single-frame transfer or the last frame of a multi-frame transfer. Otherwise, it is set to 0.
- Toggle Bit: This bit is used to verify the sequential order of consecutive frames within the same transfer. It is set to 0 for a single-frame transfer and alternates between 0 and 1 across consecutive frames in a multi-frame transfer.
- Transfer ID: This 5-bit field distinguishes each transfer and indicates its sequential order. It cycles from 0 to 31 and is used to distinguish consecutive transfers with the same CAN ID. After each transfer is completed, the Transfer ID is incremented by 1.

The transfer payload in UAVCAN is the portion of a transfer that represents the actual data content, excluding control information such as the tail byte and CRC. In a single-frame transfer, the entire transfer is contained in one CAN frame. Therefore, the portion of the data field excluding the tail byte corresponds to the transfer payload. In this case, both the SOT and EOT bits are set to 1.

In contrast, in a multi-frame transfer, the transfer payload is distributed across multiple CAN frames. The data region of each frame, excluding the tail byte, forms part of the transfer payload, whereas the transfer CRC contained in the first 2 bytes of the start frame is excluded. Accordingly, the complete transfer payload can be reconstructed by sequentially concatenating the data regions of all frames belonging to the same transfer after removing the tail byte and the transfer CRC.

### 3.3. Machine Learning Models

We selected four machine learning (ML) classifiers with different decision mechanisms—linear, bagging-based, boosting-based, and neural network-based—to assess whether the proposed features remain effective across model families [30]. The selected classifiers are random forest (RF), extreme gradient boosting (XGBoost), logistic regression (LR), and multilayer perceptron (MLP).

- Random Forest (RF): RF is an ensemble learning model that constructs multiple decision trees and produces the final output by aggregating their predictions. It provides stable classification performance while alleviating the overfitting tendency of individual decision trees.
- Extreme Gradient Boosting (XGBoost): XGBoost is an ensemble learning model based on gradient boosting, in which trees are trained sequentially to correct the errors of previously trained trees. By incorporating regularization terms, it reduces overfitting while providing high predictive performance and computational efficiency.
- Logistic Regression (LR): LR is a linear classification method that probabilistically models the relationship between input features and the output class. It estimates the probability that a given input belongs to a specific class and offers the advantages of structural simplicity and interpretability.
- Multilayer Perceptron (MLP): MLP is an artificial neural network model with one or more hidden layers between the input and output layers. The network learns nonlinear relationships through weighted connections and activation functions, enabling it to capture complex patterns among input features for classification.

## 4. Proposed Method

### 4.1. Overall Framework

The overall framework of the proposed IDS is illustrated in Figure 3, which comprises preprocessing, feature extraction, and classification stages. During preprocessing, raw UAVCAN frames are transformed into window-based inputs suitable for input to the classifiers. Frames sharing the same CAN ID are then grouped, and their tail-byte fields are parsed for subsequent feature extraction. In the feature extraction stage, 10 features are derived from each input window and grouped into payload and transfer features. The payload features are extracted from the reconstructed transfer payload data, whereas the transfer features are generated from the CAN ID and tail-byte fields to identify violations of UAVCAN transfer rules. In the classification stage, four machine learning models, namely RF, XGBoost, LR, and MLP, are used to learn patterns in normal and attack traffic and classify each input window as normal or attack.

### 4.2. Preprocessing

To capture both the UAVCAN transfer structure and the characteristics of transfer payload data, this study extracts payload and transfer features from each input window. The 29-bit CAN ID and 64-bit data field are extracted from each CAN frame to construct a 93-bit frame representation. Twenty consecutive frames are then combined into a single input window, and adjacent windows are generated using a stride of 1. To investigate the effect of the sequence length, additional experiments were conducted using sequences of 10, 20, and 30 consecutive frames. Table 1 shows that the F1-score increased from 0.9909 for 10 frames to 0.9991 for 20 frames, while the additional improvement with 30 frames was relatively small. Although the 30-frame configuration achieved the highest overall performance, it required a longer observation interval before an input window could be formed. Therefore, 20 frames were selected as a practical trade-off between detection performance and observation length. For each input window, the CAN ID and tail byte are separated, and the SOT bit, EOT bit, toggle bit, and transfer ID field are extracted from the tail byte.

The transfer payloads are then reconstructed to extract payload features, while transfer features are derived from the transfer-control information extracted from the CAN ID and tail byte. Payload features are computed from transfer payloads reconstructed according to the UAVCAN transfer structure after excluding the tail byte from each CAN data field. A single input window may contain both single-frame and multi-frame transfers, and frames from other transfers may be interleaved between the frames of a multi-frame transfer. Therefore, rather than simply concatenating payload bytes in temporal order, frames belonging to the same transfer are identified using the CAN ID, transfer ID, SOT bit, and EOT bit, and the corresponding transfer payload is reconstructed. The detailed construction of the transfer payload follows the UAVCAN transfer structure described earlier. Because a transfer may span the boundary of an input window, payload features are, in principle, computed from fully reconstructed transfer payloads. However, if no complete transfer payload is available within the window, a partially observed transfer payload is used to preserve the payload information contained in that window.

Transfer features are extracted from the CAN ID and tail-byte information by analyzing transfer ID changes and toggle-bit transitions between consecutive frames sharing the same CAN ID. The resulting counts and ratios of UAVCAN transfer-rule violations are used as transfer features. Finally, the payload and transfer features are concatenated to form a feature vector for each input window, which is used as the input to the intrusion detection model. For label assignment, each input window is labeled as an attack if it contains at least one attack frame. Accordingly, the proposed IDS performs window-level binary classification rather than frame-level or transfer-level attack localization.

Figure 4 illustrates the detailed processing workflow for payload and transfer feature extraction. Payload features are extracted by reconstructing UAVCAN transfer payloads, whereas transfer features are extracted by analyzing frame transitions between consecutive frames with the same CAN ID. The resulting six payload features and four transfer features are then concatenated into a 10-dimensional feature vector for classification.

### 4.3. Feature Extraction

As summarized in Table 2, a total of 10 features are extracted from preprocessed UAVCAN frame information and reconstructed transfer information. Because UAVCAN conveys the transfer ID, toggle bit, SOT bit, and EOT bit through the tail byte, the data characteristics at the transfer level and violations of transfer rules cannot be adequately represented using only conventional CAN frame-level fields. Accordingly, this study extracts payload features that capture the statistical characteristics of reconstructed transfer payloads and transfer features that characterize UAVCAN transfer rules. The detailed definitions of the extracted features are presented below.

#### 4.3.1. Payload Features

The payload features reflect changes in the value distribution and information content of UAVCAN transfer payloads. To this end, the byte-value distribution and entropy of the reconstructed transfer payloads are quantified to derive statistical and entropy-based features. The values calculated for each window are then aggregated to construct the payload features.

The statistical features are designed to quantify the distribution, magnitude, and repetition patterns of the byte values contained in the transfer payloads. The byte-level characteristics of each transfer payload are analyzed, and differences in transfer payload composition between normal and malicious traffic are represented as features. The three statistical payload features are denoted by $f_{1}$, $f_{2}$, and $f_{3}$, corresponding to payload_zero_ratio, payload_magnitude_mean, and payload_repeat_mean, respectively. In the equations that follow for computing the statistical payload features, $T_{i}$ denotes the *i*-th transfer payload reconstructed within window *W*, and $|T_{i}|$ denotes the number of bytes in $T_{i}$. The variable *b* represents a byte value contained in a transfer payload, where b∈{0,1,…,255}. The variable *M* denotes the total number of transfer payloads reconstructed within window *W*. In addition, $c(T_{i})$ denotes the number of occurrences of the same transfer payload pattern as $T_{i}$ within window *W*. These features are calculated as follows:

- payload_zero_ratio: This feature measures the proportion of zero-valued bytes in the transfer payloads and reflects the degree to which byte values are concentrated at zero.
  (1) $$f_{1}=\frac{1}{M}\sum_{i=1}^{M}\frac{1}{|T_{i}|}\sum_{b\in T_{i}}1\left(b=0\right)$$
- payload_magnitude_mean: This feature represents the normalized mean magnitude of the byte values contained in the transfer payloads and reflects the overall magnitude characteristics of the payload distribution.
  (2) $$f_{2}=\frac{1}{M}\sum_{i=1}^{M}\frac{1}{255|T_{i}|}\sum_{b\in T_{i}}b$$
- payload_repeat_mean: This feature measures the degree to which identical transfer payloads recur within a window, reflecting transfer payload repetition patterns.
  (3) $$f_{3}=\frac{1}{M}\sum_{i=1}^{M}1\left(c(T_{i})>1\right)$$

The entropy-based features quantify diversity based on the byte-value distribution within each transfer payload and the occurrence distribution of transfer payload patterns within a window. These features represent the information content and distributional complexity of transfer payloads that cannot be fully captured by simple value ratios or repetition indicators. Shannon entropy is therefore employed. Let *X* be a system with a finite set of possible states $x_{1},x_{2},\ldots ,x_{n}$. Then, the information entropy of system *X* is

(4) $$H\left(X\right)=-\sum_{i=1}^{n}p\left(x_{i}\right)\log_{2}p\left(x_{i}\right)$$

where $p(x_{i})$ is the probability that *X* is in state $x_{i}$.

For each reconstructed transfer payload, the system states correspond to byte values. Using the notation defined above, the number of occurrences of byte value *b* in $T_{i}$ is obtained by counting and is denoted by $C_{i}\left(b\right)$. Then, the occurrence probability of byte value *b* in $T_{i}$ can be represented as

(5) $$p_{i}\left(b\right)=\frac{C_{i}\left(b\right)}{|T_{i}|},\;b\in \left\{0,1,\ldots ,255\right\}$$

Subsequently, the Shannon entropy of the byte-value distribution in $T_{i}$ can be expressed as

(6) $$H\left(T_{i}\right)=-\sum_{b=0}^{255}p_{i}\left(b\right)\log_{2}p_{i}\left(b\right)$$

Terms for which $p_{i}\left(b\right)=0$ are omitted from the entropy calculation. Since the maximum entropy of an 8-bit byte-value distribution is $\log_{2}256=8$, $H(T_{i})$ is divided by 8 for normalization. The three entropy-based payload features are denoted by $f_{4}$, $f_{5}$, and $f_{6}$, corresponding to payload_high_entropy, payload_low_entropy, and payload_repeat_entropy, respectively. They are defined as follows:

- payload_high_entropy: This feature reflects the diversity of byte values within the transfer payloads. A larger value indicates that byte values are more evenly distributed, corresponding to greater information content and irregularity in the transfer payloads.
  (7) $$f_{4}=\frac{1}{M}\sum_{i=1}^{M}\frac{H(T_{i})}{8}$$
- payload_low_entropy: This feature reflects the degree to which byte values within the transfer payloads are concentrated in a limited set of values. A larger value indicates a simpler distribution dominated by a small number of byte values, corresponding to low diversity or repetitive byte patterns. However, short transfer payloads may produce low entropy even during normal communication. Therefore, this feature also incorporates the transfer payload length. This weighting more reliably emphasizes repetitive or highly concentrated byte distributions observed in transfer payloads of sufficient length.
  (8) $$f_{5}=\frac{1}{M}\sum_{i=1}^{M}\left(1-\frac{H(T_{i})}{8}\right)\cdot \frac{|T_{i}|}{L_{\max}}$$
  where $L_{\max}$ denotes the byte length of the longest transfer payload within window *W*. The length-weighting term reduces the influence of short transfer payloads and gives greater emphasis to entropy values obtained from transfer payloads with sufficient length.
- payload_repeat_entropy: Unlike the preceding entropy-based features, this feature is calculated from the occurrence distribution of distinct transfer payload patterns within window *W*, rather than from the byte-value distribution within each individual transfer payload. Therefore, it quantifies the diversity of transfer payload patterns within the window. A larger value indicates that distinct transfer payload patterns occur more evenly, whereas a smaller value indicates that one or a few specific transfer payload patterns occur repeatedly.
  (9) $$f_{6}=-\sum_{k=1}^{K}p\left(s_{k}\right)\log_{2}p\left(s_{k}\right)$$
  where $s_{k}$ denotes the *k*-th distinct transfer payload pattern observed within window *W*, and *K* denotes the total number of distinct patterns in that window. The occurrence probability $p(s_{k})$ of pattern $s_{k}$ is defined as
  (10) $$p\left(s_{k}\right)=\frac{C_{W}\left(s_{k}\right)}{M}$$
  where $C_{W}\left(s_{k}\right)$ denotes the number of occurrences of transfer payload pattern $s_{k}$ within window *W*.

#### 4.3.2. Transfer Features

The transfer features characterize whether UAVCAN frame transitions conform to valid transfer and tail-byte rules. To this end, the transitions of the transfer ID and toggle bit across consecutive frames with the same CAN ID are examined, and any deviation from the valid transfer construction rules is regarded as a transfer violation. The number and ratio of violations observed in each window are then quantified to construct the transfer features. The four transfer features are denoted by $f_{7}$, $f_{8}$, $f_{9}$, and $f_{10}$, corresponding to transfer_id_violation_count, transfer_id_violation_ratio, toggle_bit_violation_count, and toggle_bit_violation_ratio, respectively. They are defined as follows.

- transfer_id_violation_count: The state of each frame is categorized as one of four types: a single-frame transfer, the start frame of a multi-frame transfer, a middle frame of a multi-frame transfer, or the end frame of a multi-frame transfer. Among all possible combinations of two consecutive frame states, only those that satisfy the valid transfer-ID transition rules are regarded as normal. Conditions (a) and (b) in Table 3 describe the valid transfer-ID transitions between consecutive frames with the same CAN ID. Condition (a) applies when two consecutive frames belong to the same transfer, where the transfer ID of the subsequent frame must be identical to that of the preceding frame. Condition (b) applies when a new transfer begins, where the transfer ID of the subsequent frame must be incremented by one relative to that of the preceding transfer. Since the transfer ID is incremented modulo 32, a transfer ID of 31 is followed by 0. A pair of consecutive frames that satisfies neither condition is regarded as a transfer-ID violation. The number of such violations observed in each window is defined as transfer_id_violation_count, denoted by $f_{7}$.
- transfer_id_violation_ratio: This feature represents the proportion of consecutive frame pairs with the same CAN ID that do not satisfy the valid transfer-ID transition conditions within a window. It is calculated by dividing $f_{7}$ by the total number of consecutive frame pairs with the same CAN ID in the window, as follows:
  (11) $$f_{8}=\frac{f_{7}}{N_{\mathrm{checked}}}$$
  where $N_{\mathrm{checked}}$ denotes the number of consecutive frame pairs with the same CAN ID examined in the window. If $N_{\mathrm{checked}}=0$, the corresponding ratio is set to 0.
- toggle_bit_violation_count: Conditions (c) and (d) in Table 3 summarize the valid toggle-bit transitions. Condition (c) applies to the first frame of a transfer. The toggle bit must be 0 for both a single-frame transfer and the first frame of a multi-frame transfer. Condition (d) applies to consecutive frames within the same multi-frame transfer. In this case, the toggle bit must alternate between consecutive frames, where the alternation is expressed as $t_{n}\neq t_{p}$. Here, $t_{p}$ and $t_{n}$ denote the toggle bits of the previous and next frames, respectively. A frame pair that violates the corresponding valid toggle-bit condition is regarded as a toggle-bit violation. The number of such violations observed in each window is defined as toggle_bit_violation_count, denoted by $f_{9}$.
- toggle_bit_violation_ratio: This feature represents the proportion of consecutive frame pairs with the same CAN ID that violate the valid toggle-bit transition conditions within a window. It is calculated by dividing $f_{9}$ by the total number of consecutive frame pairs with the same CAN ID in the window, as follows:
  (12) $$f_{10}=\frac{f_{9}}{N_{\mathrm{checked}}}$$

## 5. Experiments and Analysis

### 5.1. Experimental Settings

The proposed system was implemented on a Windows 11 workstation equipped with an Intel Core i9-13900K CPU @ 3.00 GHz, 128 GB RAM, and an NVIDIA GeForce RTX 4090 GPU. To validate the effectiveness of the proposed feature extraction method, four machine learning classifiers, namely RF, XGBoost, LR, and MLP, were used. Through preprocessing and feature extraction, a feature vector was generated from each 20-frame window represented as a 20×93 binary matrix and used as the input to each classifier for binary classification of samples as normal or malicious.

The MLP was implemented using PyTorch 2.4.1, whereas RF and LR were implemented using scikit-learn, and XGBoost was implemented using the XGBoost library. For each scenario, the frames were first ordered chronologically according to their timestamps, and the continuous frame sequence was divided into non-overlapping training, validation, and test segments at ratios of 70%, 10%, and 20%, respectively. Twenty-frame windows with a stride of one were then constructed independently within each segment. Thus, although adjacent windows overlap within the same data partition, no window crosses the boundary between the training, validation, and test sets. This strategy preserves the local temporal context provided by stride-1 windowing while preventing information leakage caused by shared frames across different data partitions. For each ML classifier, the hyperparameter configuration that achieved the highest accuracy on the validation set was selected as the final setting, and the detailed settings are presented in Table 4.

### 5.2. Experimental Dataset

In this work, we used the UAVCAN dataset [31] released by the Hacking and Countermeasure Research Lab (HCRL), as summarized in Table 5. The dataset was collected using a Pixhawk 4-based UAV connected to a CAN shield and a Raspberry Pi 4. Attack messages were remotely injected into the UAVCAN bus from a PC via an SSH connection. Scenarios 1–6 contain individual flooding, fuzzy, or replay attacks, whereas Scenarios 7–10 combine two or more attack types. The scenarios differ in terms of attack type, injection interval, attack composition, and total data collection duration.

#### 5.2.1. Flooding Attack

A flooding attack is a denial-of-service (DoS) attack in which a large number of messages are repeatedly injected into the network at a high frequency, thereby occupying the available bus bandwidth. This reduces transmission opportunities for legitimate messages and may cause delays or losses in the delivery of critical control messages. In UAVCAN-based UAV environments, such disruption may interfere with motor control and the exchange of status information, thereby degrading flight stability. As a result, normal flight and mission execution may be adversely affected.

#### 5.2.2. Fuzzy Attack

A fuzzy attack injects random values into the data field to induce abnormal behavior in the receiving ECU. These random values may alter the semantics of legitimate control messages and cause the receiver to misinterpret the transmitted information. In UAVs using UAVCAN networks, such abnormal control behavior or communication errors may disrupt the normal operation of the UAV system. Consequently, flight control may become unstable and, in severe cases, critical actuator functions, including motor operation, may be impaired.

#### 5.2.3. Replay Attack

A replay attack captures legitimate messages transmitted during normal communication and retransmits them so that the receiving ECU processes them as valid messages. In UAVCAN-enabled UAV systems, when messages related to directional control or motor control are replayed, the UAV may repeatedly execute previous control commands regardless of its current state. This may destabilize flight control and, in severe cases, cause persistent movement in a particular direction or abnormal motor behavior.

### 5.3. Performance Metrics

In this study, accuracy, precision, recall, and F1-score were used to evaluate the detection performance of the proposed IDS model. These metrics are computed from the confusion matrix and quantify how effectively the classifiers distinguish between normal and attack windows. In the confusion matrix, TP denotes the number of attack windows correctly identified as attacks, TN denotes the number of normal windows correctly classified as normal, FP denotes the number of normal windows incorrectly classified as attacks, and FN denotes the number of attack windows incorrectly classified as normal. The formulas for these evaluation metrics are defined as follows:

(13) $$\mathrm{Accuracy}=\frac{\mathrm{TP}+\mathrm{TN}}{\mathrm{TP}+\mathrm{TN}+\mathrm{FP}+\mathrm{FN}}$$

(14) $$\mathrm{Precision}=\frac{\mathrm{TP}}{\mathrm{TP}+\mathrm{FP}}$$

(15) $$\mathrm{Recall}=\frac{\mathrm{TP}}{\mathrm{TP}+\mathrm{FN}}$$

(16) $$\text{F1-score}=\frac{2\times \mathrm{Precision}\times \mathrm{Recall}}{\mathrm{Precision}+\mathrm{Recall}}$$

### 5.4. Experimental Results

To evaluate the detection performance of the proposed UAVCAN-based IDS, we conducted two experiments. In the first experiment, an ablation study was performed to analyze the effectiveness of the proposed feature extraction method and the contribution of each feature set. For this purpose, three feature sets were evaluated: payload-only, transfer-only, and combined features, and their detection performance was compared. This analysis quantitatively examined the effects of payload characteristics and UAVCAN protocol-rule-based features on attack detection performance. In the second experiment, the proposed method was compared with existing CAN IDS baselines and UAVCAN-specific IDS methods. This comparison identified the limitations of the evaluated CAN IDS baselines in capturing the UAVCAN transfer structure and tail-byte-based transfer rules and demonstrated the effectiveness of the proposed UAVCAN-based feature approach.

#### 5.4.1. Ablation Analysis of Different Feature Sets

In this experiment, three feature sets were constructed to analyze the contribution of the proposed features. The first contained only payload features, the second only transfer features, and the third combined all ten features. To validate the effectiveness of the proposed feature extraction method, each feature set was evaluated using RF, MLP, XGBoost, and LR across all 10 attack scenarios in the UAVCAN dataset. This enabled us to compare the performance of the three feature sets and assess whether combining payload and transfer features improves detection performance. In addition, applying the same feature sets to multiple classifiers allowed us to examine whether the proposed features provided consistent performance across different model architectures.

The average detection performance for each feature set is presented in Table 6. When only payload features were used, most classifiers achieved high performance, indicating that the statistical and entropy-based characteristics of the transfer payload are effective for attack detection. However, LR achieved a relatively low F1-score of 90.56%, suggesting that payload features alone may be insufficient for a linear model to distinguish between normal and malicious samples.

In contrast, when only transfer features were used, all models achieved F1-scores above 99.6%, demonstrating that the UAVCAN transfer structure provides important information for attack detection. However, because these features mainly reflect violations of protocol-level transfer rules, they are limited in their ability to directly capture data-content anomalies, such as changes in payload distributions. Therefore, using payload and transfer features together enables the proposed method to capture both changes in the transfer payload and violations of transfer rules, thereby improving detection stability across various attack types.

The two feature groups capture complementary aspects of anomalous UAVCAN traffic: payload features characterize data-content changes, whereas transfer features identify violations of protocol-level transfer rules. RF obtained an F1-score of 99.92%, while MLP and XGBoost also showed high F1-scores of 99.89% and 99.88%, respectively. These results demonstrate that the combined feature set effectively captures complementary characteristics of UAVCAN attacks and maintains high detection performance across the evaluated attack categories.

The mean F1-scores of the three feature sets across the four models for each attack type are compared in Figure 5. Payload features achieved high F1-scores of 98.98% and 98.36% for flooding and fuzzy attacks, respectively, but their performance dropped to 91.91% for replay attacks. This is because replay attacks retransmit previously observed legitimate UAVCAN messages without substantially modifying their payload contents. As a result, payload-level characteristics may remain similar to those of normal traffic, which can limit the ability of payload-only features to distinguish replay attacks from normal traffic. In contrast, replayed frames are reintroduced into an ongoing UAVCAN communication sequence and may cause abnormal relationships between consecutive frames with the same CAN ID. These changes can be reflected in the proposed transfer features and used to capture anomalous transfer behavior. Therefore, transfer features provide additional protocol-level information that cannot be obtained from payload statistics alone, and the combination of the two feature groups contributes to improved replay-attack detection performance.

Accordingly, transfer features achieved an F1-score of 99.66% even for replay attacks. This indicates that the transfer features can effectively capture protocol-level changes in transfer patterns, such as transfer ID progression and frame transition behavior, even when the payload values resemble those of normal traffic. Moreover, when payload and transfer features were used together, the highest F1-scores were obtained for the evaluated attack categories. This demonstrates that using both feature groups provides consistent detection performance across the evaluated attack categories.

The detailed performance obtained for each scenario using both payload and transfer features is reported in Table 7. To assess the stability of the results, each experiment was repeated using five different random seeds (1, 10, 20, 30, and 42), and the performance is reported as the mean ± standard deviation. In the flooding and fuzzy attack scenarios, most models achieved F1-scores of approximately 99.9%, and high performance was also maintained in the replay and mixed-attack scenarios. The consistently high values of accuracy, precision, recall, and F1-score observed even in the mixed-attack scenarios indicate that the proposed feature set is not limited to a specific attack type. It also provides reliable detection performance across diverse UAVCAN attack patterns.

The false-positive rate (FPR) distributions obtained across five independent runs for each classifier and attack scenario are presented in Figure 6. Overall, the FPR remained low across most scenarios and classifiers. Relatively higher FPR values were observed in Scenarios 6, 8, and 9, particularly in replay-related scenarios. Nevertheless, the FPR remained below approximately 1.1% across all evaluated models and runs. The distributions were also narrow for most scenarios, indicating limited variation in false-positive behavior across repeated runs.

Taken together, payload features complement the transfer features by capturing attack-induced changes in payload distributions, whereas transfer features play a key role in identifying anomalies in the UAVCAN transfer structure. Therefore, the proposed approach combining the two feature groups provides the most consistent detection performance across the evaluated attack categories.

#### 5.4.2. Comparison with Existing CAN and UAVCAN IDS Methods

To further validate the effectiveness of the proposed UAVCAN-aware feature extraction method, existing in-vehicle CAN IDS models were evaluated using the same UAVCAN dataset. The results are presented in Table 8. In Table 8, Ours denotes the RF classifier trained using the proposed payload and transfer features. The proposed method outperformed the evaluated CAN IDS baselines across flooding, fuzzy, and replay attacks. The DCNN-based IDS used for comparison constructs a window of 29 consecutive CAN IDs and classifies each window into either the normal class or the attack class. This approach can effectively learn sequential changes in CAN ID patterns. However, because it relies solely on CAN IDs as input, it cannot adequately capture the transfer state and ordering information encoded in the UAVCAN tail byte. Consequently, CAN-ID-based analysis alone has limited ability to represent the protocol-level semantics of UAVCAN.

HyDL-IDS combines CNN and LSTM modules using CAN frames as input to jointly learn spatial and temporal features. Although this model may be effective for detecting pattern changes in conventional CAN traffic, it showed lower performance on replay attacks when applied to UAVCAN data. Because replay attacks retransmit previously observed legitimate UAVCAN frames, it is difficult to distinguish attack frames from normal frames using only conventional CAN-frame features based on CAN IDs and payload values.

Rec-CNN is a sequence-based CAN IDS that converts consecutive CAN ID sequences into recurrence-plot representations and uses a CNN to learn temporal pattern changes. Although this method achieved relatively high detection performance on the UAVCAN dataset, its input representation is mainly based on sequential CAN-frame information and does not explicitly reconstruct UAVCAN transfers or directly analyze protocol-specific transmission structures. Therefore, its ability to incorporate UAVCAN transfer-level information is limited. These results suggest that existing in-vehicle CAN IDS models do not sufficiently capture the transfer structure and tail-byte information of UAVCAN, highlighting the need for a dedicated IDS tailored to UAVCAN environments. In contrast, the proposed IDS explicitly exploits these UAVCAN-specific features, enabling it to achieve better detection performance than that of the evaluated CAN IDS baselines across the evaluated attack categories.

To further evaluate the effectiveness of the proposed method against UAVCAN-specific IDS approaches, a scenario-wise accuracy comparison was conducted using the results reported for WaveMamba and the Graph-based Transformer. The results are presented in Table 9. Higher accuracy than the Graph-based Transformer was achieved by the proposed method across all scenarios, while higher accuracy than WaveMamba was obtained in most scenarios. These results indicate that the proposed UAVCAN-aware feature extraction method provides competitive detection performance compared with existing UAVCAN-specific IDS approaches by explicitly incorporating transfer-level characteristics. However, because the compared results were obtained under different evaluation protocols, this comparison should be interpreted as a reference rather than a strictly controlled benchmark.

#### 5.4.3. Processing Time and Computational Cost

To evaluate the computational feasibility of the proposed IDS, feature-extraction and inference times were measured. Feature-extraction time represents the time required to generate a 10-dimensional feature vector from an in-memory 20-frame UAVCAN window, whereas inference time represents the time required for each classifier to produce a prediction from the extracted feature vector. The total processing time was calculated as the sum of these two components. As shown in Table 10, the average total processing time ranged from 1.7354 to 3.1746 ms per 20-frame window. LR achieved the shortest inference time of 0.1973 ms, whereas RF required the longest inference time of 1.6366 ms. The serialized model size was also reported as an indicator of storage requirements, ranging from 1.44 KB for LR to 1262.38 KB for RF. The reported latency represents computation time and excludes CAN-frame acquisition and communication delay. Therefore, these results should not be interpreted as direct evidence of end-to-end real-time performance on an embedded UAV platform.

### 5.5. Discussion and Limitations

This study proposed an intrusion detection system for UAVCAN environments using a feature extraction method that reflects the UAVCAN protocol structure. The proposed system extracts payload and transfer features from UAVCAN frames, and we evaluated its detection performance using various machine learning classifiers. The experimental results showed that combining payload and transfer features enabled the system to distinguish normal and malicious windows effectively. This combination also captures attack-induced changes in payload distributions and transfer-rule violations observed in UAVCAN attacks. In addition, the proposed method outperformed the evaluated in-vehicle CAN IDS baselines and showed competitive performance compared with existing UAVCAN-specific IDS approaches.

However, this study has several limitations. First, although the proposed method was validated using a publicly available UAVCAN attack dataset, the evaluation was conducted using a single dataset collected from a specific platform. Therefore, it has not yet been sufficiently demonstrated whether the proposed feature extraction framework can maintain comparable performance in other UAVCAN environments. Given the limited availability of public UAVCAN intrusion detection datasets, future work should further evaluate the generalization capability of the proposed framework using additional datasets collected from diverse UAV platforms and operational environments and, where possible, conduct cross-platform and cross-scenario evaluations. Second, the proposed IDS was designed as a binary classifier that distinguishes normal traffic from attacks. Although this configuration is suitable for attack detection, it does not provide information about the specific type of attack. Therefore, future work should extend the proposed framework to a multiclass model capable of identifying specific attack types. Third, the current evaluation is limited to flooding, fuzzy, replay, and their combined attack scenarios and therefore does not cover the full range of possible UAVCAN threats. In addition, because the proposed method is based on supervised learning, its ability to detect previously unseen attack types is limited. To address this issue, threshold-based anomaly detection, unsupervised learning, or semi-supervised learning methods could be integrated to improve the detection of unknown and previously unobserved attacks. Finally, the evaluated attacks may introduce abnormal changes in UAVCAN transfer behavior, which can be effectively captured by the proposed transfer features. However, the effectiveness of these features may decrease when an attacker deliberately preserves normal UAVCAN transfer rules while conducting an attack. Therefore, future work should evaluate the proposed method under more diverse attack conditions, including adaptive attacks that remain compliant with UAVCAN protocol rules.

## 6. Conclusions

This study presented a protocol-aware intrusion detection framework for UAVCAN networks. The framework reconstructs UAVCAN transfers and combines payload-level statistical and entropy-based features with transfer-rule features derived from the tail byte. The ablation results showed that transfer features were highly effective for identifying replay attacks, for which payload-only features were less reliable, while the combined feature set provided the most consistent performance across the ten evaluated scenarios. The best-performing configuration achieved average accuracy, precision, and recall of 99.89%, 99.96%, and 99.88%, respectively, outperforming the evaluated automotive CAN IDS baselines and achieving competitive performance compared with existing UAVCAN-specific IDS methods. These results highlight the benefit of incorporating UAVCAN-specific transfer semantics into intrusion detection. Future work will evaluate cross-platform and cross-scenario generalization using additional UAVCAN datasets, investigate protocol-compliant and adaptive attacks, and extend the framework to multiclass classification and previously unseen attack detection.

## Figures and Tables

**Figure 1:**
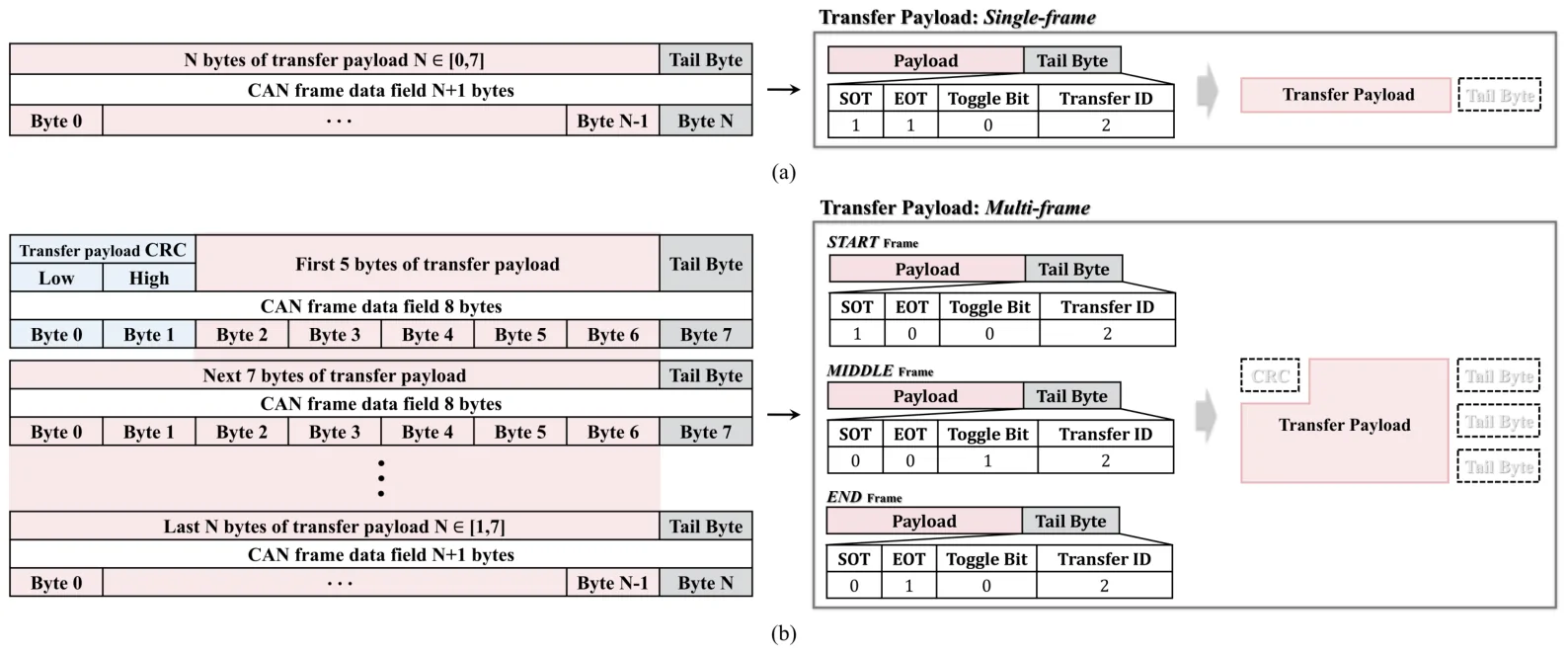
UAVCAN transfer structures over Classical CAN: (**a**) single-frame transfer and (**b**) multi-frame transfer. A single-frame transfer carries up to 7 bytes of transfer payload and one tail byte, whereas a multi-frame transfer distributes the payload across multiple CAN frames.

**Figure 2:**
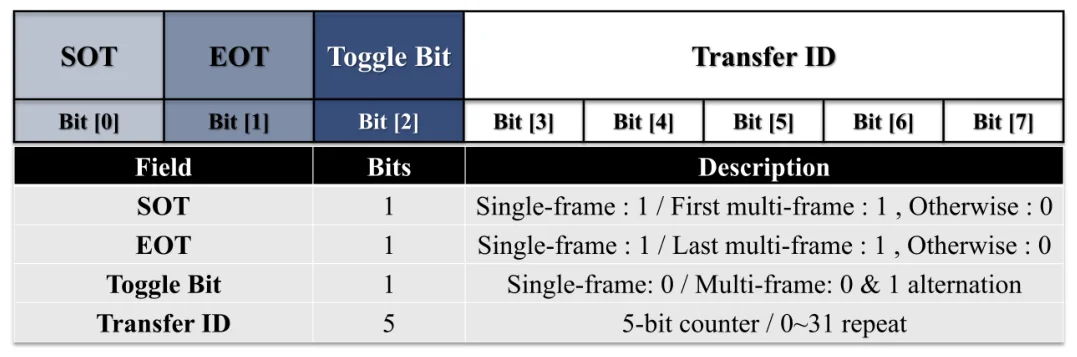
Format of the UAVCAN tail byte, consisting of the SOT, EOT, toggle-bit, and 5-bit transfer-ID fields.

**Figure 3:**
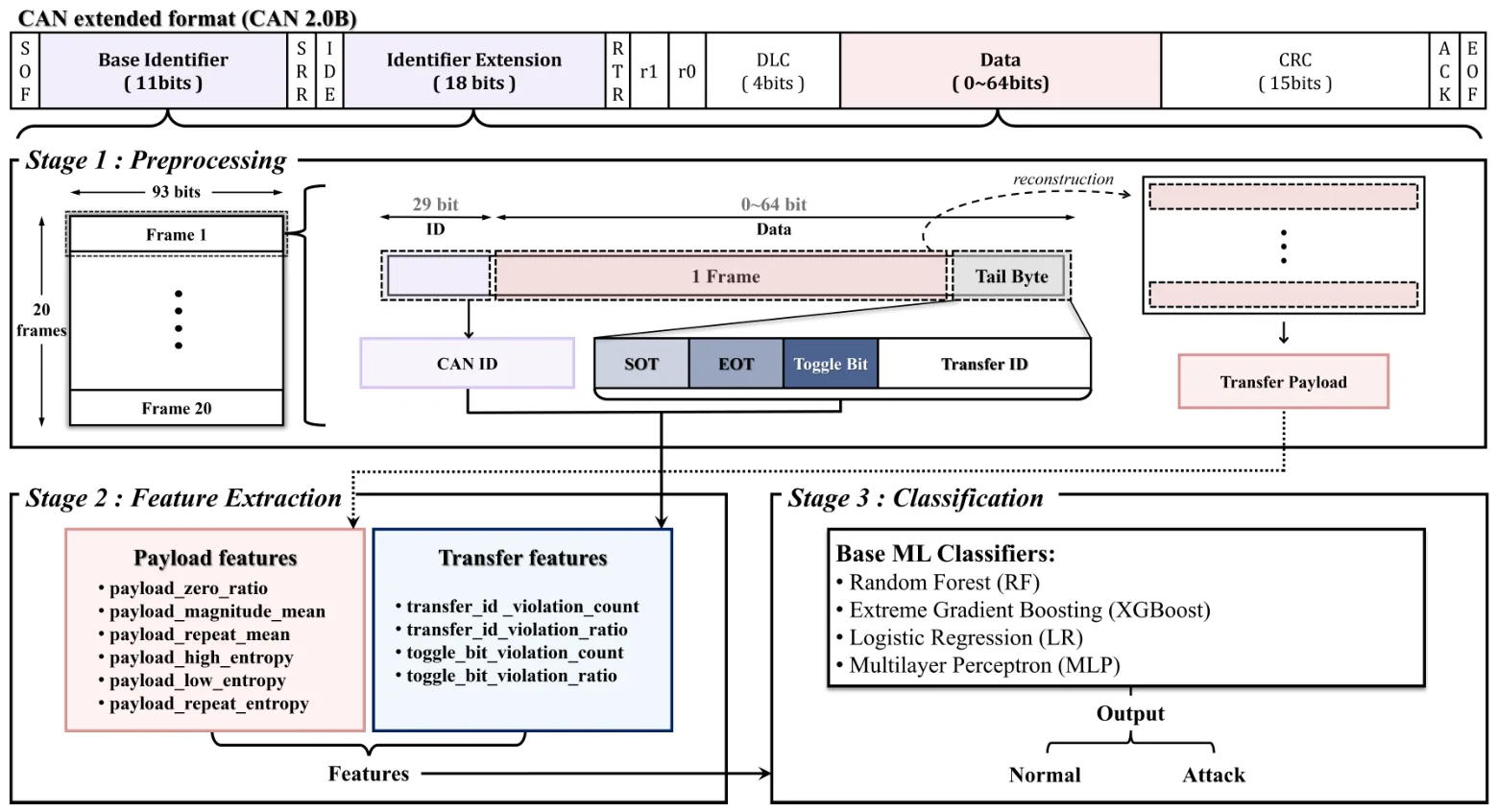
Overview of the proposed IDS framework.

**Figure 4:**
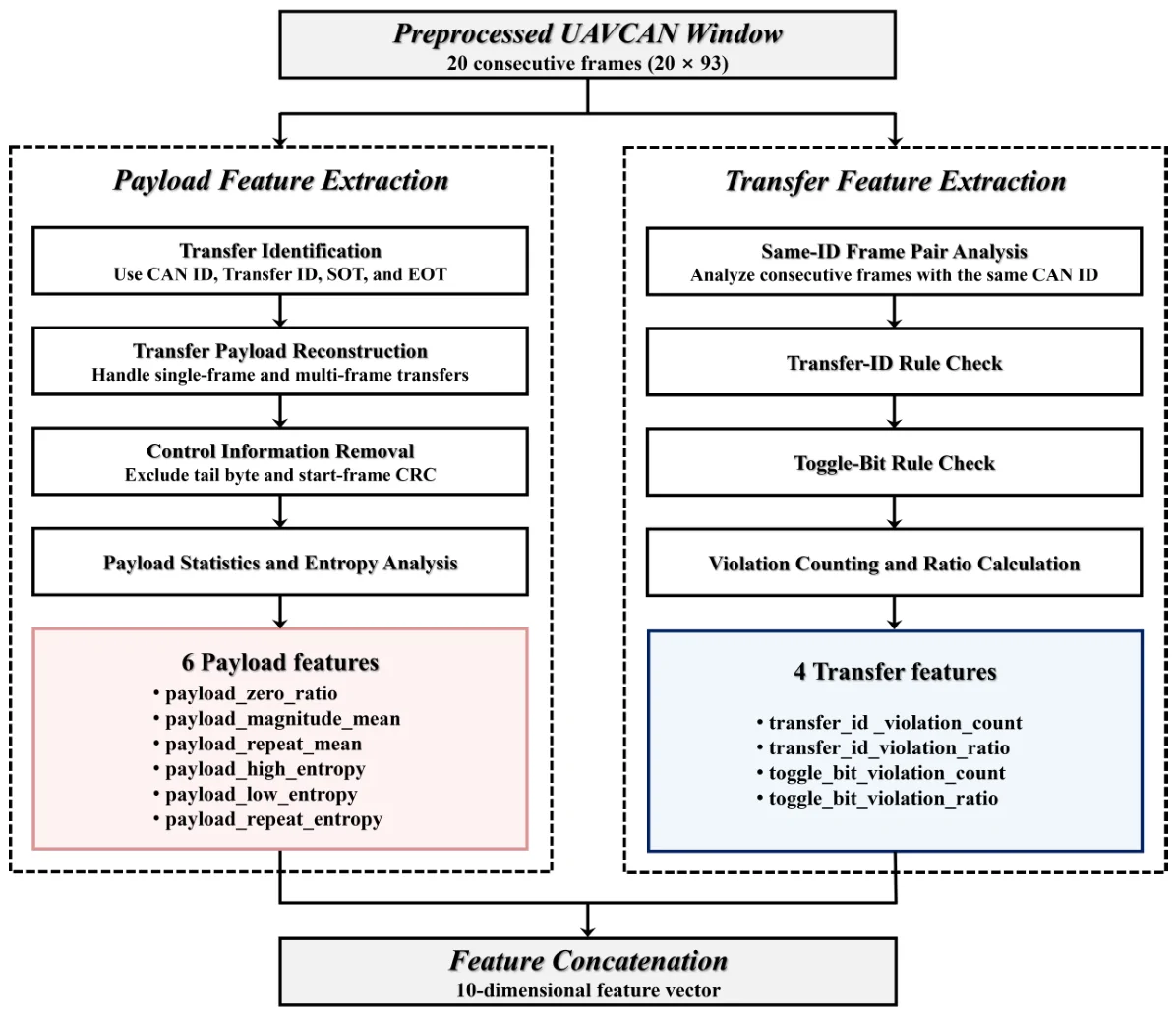
Detailed flowchart of payload and transfer feature extraction.

**Figure 5:**
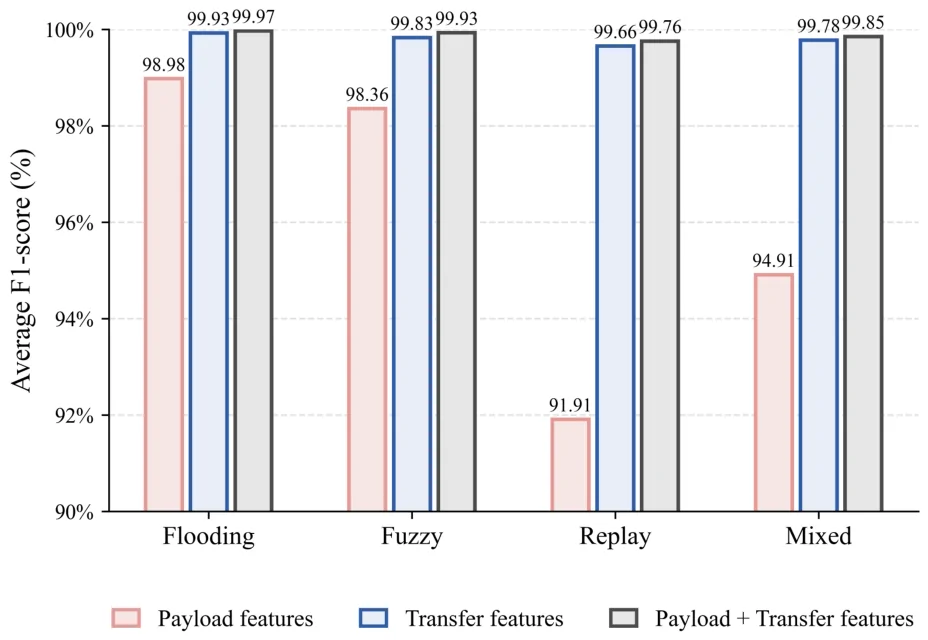
Average F1-score by feature set and attack type.

**Figure 6:**
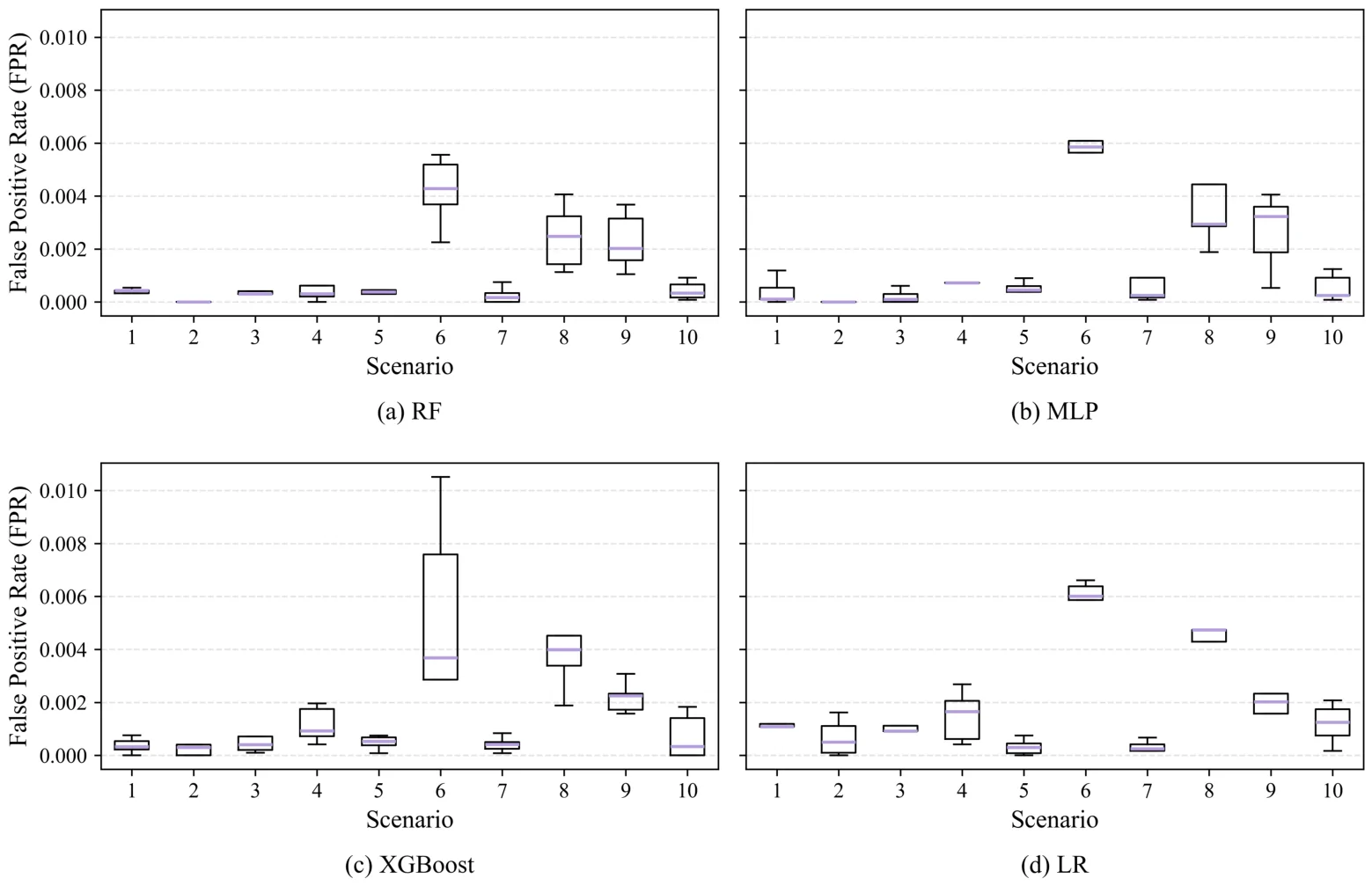
Distribution of false-positive rates (FPRs) across five independent runs for (**a**) RF, (**b**) MLP, (**c**) XGBoost, and (**d**) LR in each UAVCAN attack scenario.

**Table 1:** Detection performance for different input window sizes.

Window Size	Accuracy	Precision	Recall	F1-Score
10 frames	0.9875	0.9930	0.9888	0.9909
20 frames	0.9988	0.9994	0.9989	0.9991
30 frames	0.9992	0.9996	0.9992	0.9994

**Table 2:** Proposed feature set for the IDS.

Group	Index	Feature	Description
Payload	$f_{1}$	payload_zero_ratio	Average proportion of zero bytes in the transfer payload within a window
	$f_{2}$	payload_magnitude_mean	Average normalized transfer payload magnitude within a window
	$f_{3}$	payload_repeat_mean	Average repetition ratio of transfer payload patterns within a window
	$f_{4}$	payload_high_entropy	Mean normalized Shannon entropy of byte-value distributions across reconstructed transfer payloads
	$f_{5}$	payload_low_entropy	Mean length-weighted complement of normalized payload entropy
	$f_{6}$	payload_repeat_entropy	Shannon entropy of the transfer-payload pattern occurrence distribution within a window
Transfer	$f_{7}$	transfer_id_violation_count	Number of same-ID consecutive frame pairs violating the transfer ID transition rule
	$f_{8}$	transfer_id_violation_ratio	Ratio of same-ID consecutive frame pairs violating the transfer ID transition rule
	$f_{9}$	toggle_bit_violation_count	Number of same-ID consecutive frame pairs violating the toggle bit transition rule
	$f_{10}$	toggle_bit_violation_ratio	Ratio of same-ID consecutive frame pairs violating the toggle bit transition rule

**Table 3:** Valid UAVCAN frame-transition rules used for transfer-feature extraction.

(**a**) **Same Transfer**					
**Case**	**Previous Frame**		**Next Frame**		**Next** **Transfer ID**
	**SOT**	**EOT**	**SOT**	**EOT**	
1	1	0	0	0	Same
2	1	0	0	1	Same
3	0	0	0	0	Same
4	0	0	0	1	Same
(**b**) **Different Transfer**					
**Case**	**Previous Frame**		**Next Frame**		**Next** **Transfer ID**
	**SOT**	**EOT**	**SOT**	**EOT**	
1	1	1	1	1	+1
2	1	1	1	0	+1
3	0	1	1	1	+1
4	0	1	1	0	+1
(**c**) **Initial Toggle Rule**					
**Case**	**Current Frame**				**Toggle** **Bit**
	**SOT**		**EOT**		
1	1		1		0
2	1		0		0
(**d**) **Toggle Alternation**					
**Case**	**Previous Frame**		**Next Frame**		**Toggle** **Rule**
	**SOT**	**EOT**	**SOT**	**EOT**	
1	1	0	0	0	$t_{n}\neq t_{p}$
2	1	0	0	1	$t_{n}\neq t_{p}$
3	0	0	0	0	$t_{n}\neq t_{p}$
4	0	0	0	1	$t_{n}\neq t_{p}$

**Table 4:** Hyperparameter settings of the four ML classifiers.

Group	Classifier	Hyperparameter Setting
Traditional ML	Random Forest	n_estimators = 30, max_features = sqrt
	XGBoost	n_estimators = 30, max_depth = 5
	Logistic Regression	C = 0.3, solver = liblinear
Neural Network	MLP	hidden layers = 256–128, ReLU, AdamW, lr = 0.001, batch size = 32, epochs = 50, dropout = 0.3, weight decay = $10^{-4}$, patience = 10

**Table 5:** UAVCAN attack scenarios used in the experiments.

Scenario	Attack Type	Attack InjectionInterval (s)	Duration (s)	Normal Frames/Attack Frames
1	Flooding	0.0015	180	91,042/116,816
2	Flooding	0.0050	180	102,240/31,930
3	Fuzzy	0.0015	180	101,601/95,878
4	Fuzzy	0.0050	180	104,204/29,170
5	Replay	0.0050	210	129,996/50,612
6	Replay	0.0050	280	160,233/81,088
7	Flooding + Fuzzy	0.0050	240	141,550/92,612
8	Fuzzy + Replay	0.0050	240	150,492/115,308
9	Flooding + Replay	0.0050	270	163,126/67,252
10	Flooding + Fuzzy + Replay	0.0050	220	131,530/75,850

**Table 6:** Average detection performance by feature set.

Feature Set	Model Type	Accuracy	Precision	Recall	F1-Score
Payload Features	RF	0.9669	0.9770	0.9755	0.9762
	MLP	0.9700	0.9801	0.9765	0.9783
	XGBoost	0.9620	0.9763	0.9687	0.9725
	LR	0.8615	0.8815	0.9359	0.9056
Transfer Features	RF	0.9971	0.9995	0.9965	0.9980
	MLP	0.9971	0.9994	0.9964	0.9980
	XGBoost	0.9971	0.9994	0.9965	0.9980
	LR	0.9970	0.9993	0.9964	0.9978
Payload + Transfer Features	RF	0.9989	0.9996	0.9988	0.9992
	MLP	0.9985	0.9994	0.9985	0.9989
	XGBoost	0.9982	0.9994	0.9981	0.9988
	LR	0.9972	0.9993	0.9967	0.9980

**Table 7:** Scenario-wise detection performance using combined payload and transfer features. Values are reported as mean ± standard deviation over five independent runs.

Attack	Model	Accuracy	Precision	Recall	F1-Score
Flooding [Scenario 1]	RF	0.9999±0.0000	0.9999±0.0000	1.0000±0.0000	0.9999±0.0000
	MLP	0.9998±0.0001	0.9999±0.0001	0.9999±0.0001	0.9999±0.0000
	XGBoost	0.9996±0.0001	0.9999±0.0001	0.9996±0.0001	0.9997±0.0001
	LR	0.9992±0.0000	0.9997±0.0001	0.9993±0.0001	0.9995±0.0000
Flooding [Scenario 2]	RF	0.9999±0.0001	0.9999±0.0002	1.0000±0.0000	1.0000±0.0001
	MLP	0.9999±0.0000	1.0000±0.0000	0.9999±0.0000	0.9999±0.0000
	XGBoost	0.9999±0.0001	0.9999±0.0001	0.9999±0.0001	0.9999±0.0001
	LR	0.9985±0.0001	0.9996±0.0004	0.9979±0.0003	0.9988±0.0001
Fuzzy [Scenario 3]	RF	0.9998±0.0000	0.9999±0.0001	0.9999±0.0001	0.9999±0.0000
	MLP	0.9999±0.0001	0.9999±0.0001	0.9999±0.0001	0.9999±0.0000
	XGBoost	0.9995±0.0001	0.9999±0.0001	0.9994±0.0001	0.9996±0.0000
	LR	0.9988±0.0002	0.9997±0.0001	0.9987±0.0002	0.9992±0.0001
Fuzzy [Scenario 4]	RF	0.9998±0.0001	0.9998±0.0002	0.9998±0.0001	0.9998±0.0001
	MLP	0.9995±0.0002	0.9996±0.0002	0.9996±0.0002	0.9996±0.0001
	XGBoost	0.9990±0.0003	0.9993±0.0004	0.9990±0.0004	0.9992±0.0002
	LR	0.9966±0.0004	0.9991±0.0006	0.9955±0.0006	0.9973±0.0003
Replay [Scenario 5]	RF	0.9992±0.0001	0.9998±0.0001	0.9990±0.0002	0.9994±0.0001
	MLP	0.9992±0.0002	0.9997±0.0002	0.9990±0.0003	0.9993±0.0002
	XGBoost	0.9992±0.0000	0.9997±0.0002	0.9990±0.0002	0.9993±0.0000
	LR	0.9988±0.0002	0.9998±0.0002	0.9983±0.0003	0.9991±0.0002
Replay [Scenario 6]	RF	0.9956±0.0003	0.9984±0.0005	0.9955±0.0004	0.9969±0.0002
	MLP	0.9942±0.0006	0.9979±0.0006	0.9941±0.0014	0.9960±0.0004
	XGBoost	0.9942±0.0006	0.9979±0.0013	0.9941±0.0005	0.9960±0.0004
	LR	0.9922±0.0006	0.9978±0.0005	0.9914±0.0012	0.9946±0.0004
Flooding + Fuzzy [Scenario 7]	RF	0.9999±0.0001	0.9999±0.0001	1.0000±0.0000	0.9999±0.0000
	MLP	0.9998±0.0001	0.9998±0.0001	0.9999±0.0000	0.9999±0.0001
	XGBoost	0.9995±0.0001	0.9999±0.0001	0.9995±0.0001	0.9997±0.0000
	LR	0.9992±0.0001	0.9999±0.0001	0.9990±0.0001	0.9994±0.0001
Fuzzy + Replay [Scenario 8]	RF	0.9979±0.0002	0.9992±0.0004	0.9980±0.0004	0.9986±0.0001
	MLP	0.9971±0.0003	0.9987±0.0007	0.9974±0.0006	0.9981±0.0002
	XGBoost	0.9968±0.0002	0.9988±0.0004	0.9970±0.0005	0.9979±0.0001
	LR	0.9958±0.0003	0.9985±0.0004	0.9959±0.0005	0.9972±0.0002
Flooding + Replay [Scenario 9]	RF	0.9969±0.0002	0.9991±0.0004	0.9966±0.0005	0.9978±0.0001
	MLP	0.9963±0.0002	0.9989±0.0006	0.9959±0.0007	0.9974±0.0001
	XGBoost	0.9956±0.0002	0.9991±0.0002	0.9947±0.0005	0.9969±0.0001
	LR	0.9943±0.0003	0.9991±0.0003	0.9928±0.0004	0.9960±0.0002
Flooding + Fuzzy + Replay [Scenario 10]	RF	0.9996±0.0001	0.9998±0.0001	0.9996±0.0002	0.9997±0.0001
	MLP	0.9993±0.0002	0.9998±0.0002	0.9992±0.0005	0.9995±0.0002
	XGBoost	0.9992±0.0002	0.9997±0.0003	0.9992±0.0003	0.9994±0.0001
	LR	0.9986±0.0002	0.9995±0.0003	0.9985±0.0005	0.9990±0.0002

**Table 8:** Performance comparison of the proposed method with existing CAN IDS baselines. The reported values are averages over Scenarios 1–2 for flooding attacks, Scenarios 3–4 for fuzzy attacks, and Scenarios 5–6 for replay attacks.

Attack Type	Method Group	Model	Precision	Recall	F1-Score
Flooding	Existing CAN IDS	DCNN [16]	0.8884	0.9412	0.9140
		HyDL-IDS [24]	0.9708	1.0000	0.9852
		Rec-CNN [23]	0.9640	0.9737	0.9688
	Proposed Method	RF	0.9999	1.0000	0.9999
		MLP	0.9999	0.9999	0.9999
		XGBoost	0.9999	0.9997	0.9998
		LR	0.9996	0.9986	0.9991
Fuzzy	Existing CAN IDS	DCNN [16]	0.8612	0.9410	0.8993
		HyDL-IDS [24]	0.9377	0.9869	0.9617
		Rec-CNN [23]	0.9630	0.9770	0.9700
	Proposed Method	RF	0.9998	0.9999	0.9999
		MLP	0.9998	0.9997	0.9997
		XGBoost	0.9996	0.9992	0.9994
		LR	0.9994	0.9971	0.9982
Replay	Existing CAN IDS	DCNN [16]	0.8534	0.9482	0.8983
		HyDL-IDS [24]	0.8636	0.8201	0.8413
		Rec-CNN [23]	0.9886	0.9652	0.9768
	Proposed Method	RF	0.9991	0.9972	0.9982
		MLP	0.9988	0.9965	0.9977
		XGBoost	0.9988	0.9965	0.9977
		LR	0.9988	0.9948	0.9968

**Table 9:** Scenario-wise accuracy comparison with existing UAVCAN-specific IDS methods. The results for WaveMamba and Graph-based Transformer are based on the values reported in WaveMamba [28].

Scenario	WaveMamba	Graph-Based Transformer	Ours
Scenario 1	0.9870	0.9940	0.9999
Scenario 2	0.9990	0.9670	0.9999
Scenario 3	0.9970	0.9850	0.9998
Scenario 4	0.9980	0.9990	0.9998
Scenario 5	0.9790	0.8430	0.9992
Scenario 6	0.9910	0.9450	0.9956
Scenario 7	0.9970	0.9170	0.9999
Scenario 8	0.9980	0.9340	0.9979
Scenario 9	0.9800	0.8160	0.9969
Scenario 10	0.9880	0.8990	0.9996

Note: WaveMamba and Graph-based Transformer were evaluated on the HCRL UAVCAN Attack Dataset. Ours denotes the RF classifier using the combined payload and transfer features.

**Table 10:** Processing latency and serialized model size of the evaluated classifiers.

Model	Feature Extraction (ms/Window)	Inference (ms/Window)	Total Processing (ms/Window)	Serialized Model Size (KB)
RF	1.5381	1.6366	3.1746	1262.38
MLP	1.5381	0.3459	1.8840	142.70
XGBoost	1.5381	0.3181	1.8561	44.99
LR	1.5381	0.1973	1.7354	1.44

## Data Availability

The dataset analyzed in this study is publicly available from the HCRL UAVCAN Attack Dataset at https://ocslab.hksecurity.net/Datasets/uavcan-attack-dataset (accessed on 25 August 2026).
